# Burnstock oration — purinergic signalling in kidney transplantation

**DOI:** 10.1007/s11302-022-09865-3

**Published:** 2022-04-26

**Authors:** Karen M. Dwyer

**Affiliations:** School of Medicine, Deakin University, Geelong, 3220 Australia

**Keywords:** CD39, ATP, Adenosine receptors, Regulatory T cells, Treg, Ischaemia reperfusion injury

## Abstract

Kidney transplantation is the preferred treatment for individuals with kidney failure offering improved quality and quantity of life. Despite significant advancements in short term graft survival, longer term survival rates have not improved greatly mediated in large by chronic antibody mediated rejection. Strategies to reduce the donor kidney antigenic load may translate to improved transplant survival. CD39 on the vascular endothelium and on circulating cells, in particular regulatory T cells (Treg), is upregulated in response to hypoxic stimuli and plays a critical role in regulating the immune response removing proinflammatory ATP and generating anti-inflammatory adenosine. Herein, the role of CD39 in reducing ischaemia–reperfusion injury (IRI) and on Treg within the context of kidney transplantation is reviewed.

‘*I think scientists are creative people exactly like artists. I am going to treat you like an artist. I want to let you express your creative spirit in whatever way suits you best.*’ [extract from 1].

I was humbled and honoured to receive the 2021 Burnstock Oration in recognition of outstanding contributions to purinergic signalling. I had the privilege of speaking with Prof Burnstock during one of his stays in Melbourne — his vitality, vivaciousness, interest, congeniality and superb intellect are vivid memories of this meeting. Vale Professor Geoffrey Burnstock.

I dedicate this award to my mentors and sponsors Professors Tony d’Apice, Simon Robson and Terry Strom who have guided me on my journey in purinergic signalling in kidney transplantation. Tony supervised my PhD studies investing time in me, a novice in CD39 biology and science in general. I am grateful for the wisdom he bestowed, his confidence in my strengths and abilities and encouragement to pursue an academic career. I completed my post doc studies with Simon and Terry, who opened my eyes to wonders of international research, facilitated creative thinking and epitomised leadership. Simon remains a constant source of advice and perspective. I am indebted to Professor Silvia Deaglio for her comradery, collegiality, agile intellect, scientific analysis, collaboration, and friendship during our post doc studies in Boston.

“*A mentor is not someone who walks ahead of us and tells us how they did it.*

*A mentor is someone who walks alongside us to guide us on what we can do.*” *Simon Sinek.*

## CD39: the master switch

The dual function of adenosine triphosphate (ATP) as a signalling molecule was first documented in 1962 by Prof Burnstock himself and proclaimed a decade later, almost 50 years after the initial characterisation of ATP as the energy currency of all living cells [reviewed in 2]. Whilst existing in the intracellular space at high concentration, the negative charge of ATP restricts movement across the cell membrane and thus it is only found in micromolar concentrations outside the cells under physiological conditions. In pathological states however, ATP may be extruded from necrotic cells or released during apoptosis in a regulated manner via pannexin hemichannels [3]. Furthermore, endothelial cells and activated inflammatory cells can also release ATP through connexin hemichannels [4]. With the identification of an array of P2X and P2Y receptors and their distribution throughout the body, it has become clear that the biological effects of ATP are diverse and pervasive. In the context of kidney transplantation, ATP in the most part promotes an inflammatory and thrombotic response through a direct action on the kidney endothelium and systemically via activation of inflammatory cells and platelets [4].

The pathophysiological effects of ATP are tightly regulated by the family of ecto-nucleoside triphosphate diphosphohydrolases (NTPDases) such as CD39, which rapidly hydrolyse ATP. ATP is hydrolysed to adenosine diphosphate (ADP), which is in turn hydrolysed to adenosine monophosphate (AMP). AMP is degraded to adenosine by CD73. Extracellular adenosine signals through four G-protein coupled P1 receptors which in general oppose the actions of ATP. Furthermore, adenosine via equilibrative nucleoside transporter (ENT) 1 and 2 can move back into the intracellular space, where it is degraded to inosine (via adenosine deaminase) or re-phosphorylated to AMP (via adenosine kinase) [4].

CD39 (also known as NTPDase 1) is the prototype of the four membrane — bound NTPDases and hydrolyses ATP and ADP with equal potency. CD39L1 (also known as NTPDase 2) preferentially hydrolyses ADP over ATP. NTPDase 3 and NTPDase 8 preferentially hydrolyse ATP over ADP [5]. Whilst the CD39-adenosine axis is the predominant purinergic pathway in renal pathophysiology and immune regulation, there are several other enzymes that impact ATP regulation which has been discussed recently [6] and is beyond the focus of this review.

CD39 is expressed widely within the immune system and on the vascular endothelium. In the kidney, CD39 is expressed on vascular endothelium and within the collecting ducts in both the cortex and the medulla. It is also expressed in the ascending thin limb of the loop of Henle [7, 8] and the glomerulus [8]. CD73 is predominantly expressed in the glomeruli, on the endothelium and peritubular fibroblasts [9]. Within the kidney, the enzymatic efficiency of CD73 is less than that of CD39 [10] resulting in ATP being degraded more rapidly than adenosine is generated, which highlights the importance of both the removal of nucleotides and production of adenosine in kidney disease.

Adenosine is the canonical ligand of the four G-protein-coupled P1 receptors known as the A_1_, A_2A_, A_2B_ and A_3_ receptors. Adenosine receptors are classified based on their differential coupling to adenylyl cyclase to regulate cyclic adenosine monophosphate (cAMP) levels: A_1_ and A_3_ receptors are coupled to the G-inhibitory subunit and the activation leads to a reduction in the level of intracellular cAMP, whereas as A_2A_ and A_2B_ receptors are coupled to the G-stimulatory subunit and their activation results in an increase in intracellular cAMP. Signalling through these receptors is in fact far more complex and adenosine receptors can also modulate a variety of additional second messengers. For example, the A_2B_ receptor also couples to Gq proteins, which stimulate phospholipase C activity and intracellular calcium mobilisation [11, 12]. The A_2B_ receptor has lower affinity and thus high pericellular concentrations of adenosine, as seen in pathological states, are required compared to the other P1 receptors [12]. Furthermore, the A_2B_ receptor has also been shown to interact with AMP via computational modelling, in vitro assays [13] and in vivo studies [14]. An additional layer of complexity exists with receptor dimerization which may be necessary for full receptor functionality. For example, heterodimerization of the A_2A_ and A_2B_ receptors is necessary for the A_2B_ receptor to be efficiently expressed on the cell surface in vitro [15].

The ATP — CD39 — adenosine axis functions as a dynamic pathway [16] that regulates the availability of ATP and adenosine for P2 and P1 receptor activation respectively in the extracellular milieu. CD39 thus has profound implications in kidney transplantation outcomes, in the most part through modifying the antigenicity of the donor kidney and thus the recipient immune response. Antibody mediated rejection is the leading cause of kidney failure following transplantation [17]. Whilst many factors that increase the risk of antibody mediated rejection are non-modifiable, such as donor and recipient age [18], others such as ischaemia reperfusion injury (IRI) [19] present an opportunity for manipulation. Furthermore, regulatory T cells (Treg) are central to preventing kidney transplant rejection and, in animal models, are a potential therapeutic target [20]. This review will focus on the CD39 — adenosine axis in models of kidney IRI and on Treg.

## Ischaemia reperfusion injury (IRI)

IRI is an obligatory insult in the kidney transplantation process occurring with organ procurement and engraftment. Warm ischaemia occurs in the context of organ procurement; cold ischaemia uniquely occurs in transplantation where the donor organ undergoes cold preservation, which can be prolonged for many hours. The re-establishment of blood flow at the time of engraftment paradoxically incites further inflammation despite terminating hypoxic injury. The clinical implications of IRI include systemic inflammatory effects, organ dysfunction encompassing delayed graft function, acute allograft rejection and chronic allograft nephropathy.

Purinergic signalling has been implicated in both the pathogenesis of, and the immune response to, IRI [21]. Hypoxic injury causes a dramatic rise in P2X7 receptor expression [22] and the pericellular concentration of ATP (released from injured or dying cells), exaggerated by the loss of CD39 and CD73 from activated endothelial cells [23], which ignites an inflammatory response. Indeed, mice deficient in *Cd39* are highly susceptible to kidney injury [24]. Conversely, mice that over-express human CD39 [25] are robustly protected in models of both warm [26] and cold ischaemia [27] an effect that persists whether CD39 over-expression is restricted to the vascular endothelium or the circulating cells. CD39 activity can be augmented pharmacologically with soluble CD39 [26, 28] given prior to ischaemia which also mitigated injury. Such an effect can be simulated through the process of ischaemic preconditioning whereby repeated short episodes of ischaemia (4 min) are followed by short periods of reperfusion (4 min) prior to extended ischaemia and reperfusion [29]. Ischaemic preconditioning resulted in an increase in CD39 transcript expression and pericellular adenosine concentration [29] mediated via specificity protein 1 (Sp1) [30], a ubiquitously expressed transcription factor implicated in promoting hypoxic gene transcription. Intriguingly protection from IRI is conferred with inhaled carbon monoxide, an effect that is dependent on the induction of CD39 expression, within 2 h of reperfusion and which is ineffective in mice deficient in *Cd39* [31]. This effect was mediated through upregulation and stabilisation of circadian rhythm protein Period 2 (Per2) and serum erythropoietin (EPO) [31].

Whilst ATP promoted kidney IRI predominantly via the P2X7 receptor [22], an effect which can be abrogated by administration of a P2X7 receptor antagonist up to 6 h post reperfusion [32], CD39-adenosine protection is mediated via A_1_, A_2A_ and A_2B_ receptors depending on the predominant cell type involved [reviewed in 33]. A_1_ receptor activation on proximal tubular cells ameliorated ischemic-induced acute kidney injury with reduced apoptosis, necrosis and inflammation [34, 35]. Stimulation of the A_2A_ receptor on circulating CD4^+^ T cells mediated protection from IRI, an effect that was IFNγ dependent [36]. Additional studies have shown that A_2A_ receptor activation on Treg and [37] and dendritic cells [38] protected against kidney IRI. Treg play a crucial role in IRI — depletion of Treg worsened IRI whereas the infusion of Treg enhanced the repair response [reviewed in 39]. On the vascular endothelium, the A_2B_ receptor was upregulated within 24 h [31, 40] and played a predominant role in mediating protection against kidney IRI [41].

Whilst much of the impact of CD39 has been attributed to the removal of ATP and generation of adenosine, AMP is a necessary intermediary. Our work has shown that AMP concentrations increased immediately following kidney ischaemia [26] and mice treated with a CD73 inhibitor or deficient in CD73 are paradoxically protected in a model of mild kidney IRI [42], an effect mediated by the A_2B_ receptor [14].

In addition to increasing the immunogenicity of the transplanted kidney, IRI portends to chronic kidney disease which impacts graft function. Purinergic signalling again plays a significant role in chronic kidney disease which is characterised histologically by kidney fibrosis. The fibroblast expresses CD73 and A_2B_ receptor [43] and agonism of A_2B_ receptor increased transcription of profibrotic and inflammatory mediators [44]. Indeed, CD73 and A_2B_ receptor [45] and IL-6 [46] were significantly elevated in the kidneys of individuals with chronic kidney disease and further elevated in those with hypertension. So, whilst a transient increase in CD39 protects acutely and prevents chronic kidney disease, the sustained generation of adenosine through the concerted and persistent action of CD39 and CD73 leads to increased myofibroblast activity and fibrosis [26, 47].

## Regulatory T cells (Treg)

Whilst several studies have shown a positive correlation between the presence of Treg and clinical outcomes in kidney transplantation [48, 49], the function, phenotype and biology of Treg continue to evolve [reviewed in 50]. Initially described by using solely the intensity of CD25 expression to identify CD4^+^CD25^hi^ Treg, a significant advancement came with the identification of the transcription factor FOXP3 necessary for Treg survival and suppressive function. Further differentiation markers have been identified including CD39 [51, 52], which is of functional significance facilitating adenosine generation and, via A_2A_ receptor, halting cellular proliferation [52, 53]. Indeed, Treg from mice lacking *Cd39* have suboptimal suppressive capacity and resulted in more rapid rejection of allogenic skin grafts [52] and the spontaneous development of autoimmune diseases associated with Th1 immune deviation [54].

In humans, four T cell populations can be defined by the differential expression of CD4, CD25 and CD39. Notably, unlike in mice, human Treg cells do not express CD73, implicating a paracrine mechanism for the generation of adenosine whereby CD73 is expressed on target cells [55]. CD39 expression on Treg is increased by a positive feedback loop, in which the *Cd39* promoter is transactivated by adenosine [56]. CD39 expression also decreases Treg cell susceptibility to ATP-induced cell death [57]. The presence of CD39 denoted memory cells (effector (mTeff) and regulatory (mTreg) in 1:1) [55] whereas the absence of CD39 on CD4^+^CD25^+^ cells predicted cells with Th17 potential [55, 58]. Using these markers, T cell number and dynamics can be tracked before and after transplantation and in the setting of stable graft function remain static [55]. However, the kinetics of Treg differ and in the presence of stable graft function, the immunosuppressive capacity of CD4^+^CD25^+^CD39^+^ Treg was enhanced compared to healthy controls [59]. This was even more apparent in individuals deemed tolerant. Here, memory Treg were increased, had greater suppressive function and CD39 expression and an enhanced capacity to degrade extracellular ATP compared to those with stable function [60, 61]. These data contrast with the situation of antibody-mediated rejection characterised by inflammation in which the effector: memory T cell ratio is heavily skewed to effector cells [55].

Since the original description of CD39 on Treg [51, 52], CD39 has been identified on T regulatory type 1 cells (Tr1) cells — a subset of Treg characterised by the secretion of IL-10 and the lack of FOXP3 expression. CD39 was essential for the full suppressive activity of Tr1 cells in vitro and in vivo [62] and in human kidney transplantation, the numbers of Tr1 cells were positively correlated with stable graft function [63]. Moreover, CD39 promoted Tr1 cell differentiation by limiting extracellular ATP [62]. Another T cell subset with suppressive capabilities includes suppressive Th17 cells that have high levels of CD39, co-express CD73 and generate adenosine [64]. These CD39 expressing subsets have non-redundant roles in experimental mouse model of autoimmune encephalomyelitis [62] and in humans with Crohn’s disease [64]. A role for suppressive Th17 cells in kidney transplantation however is yet to be defined.

## Conclusion

**Fig. 1:**
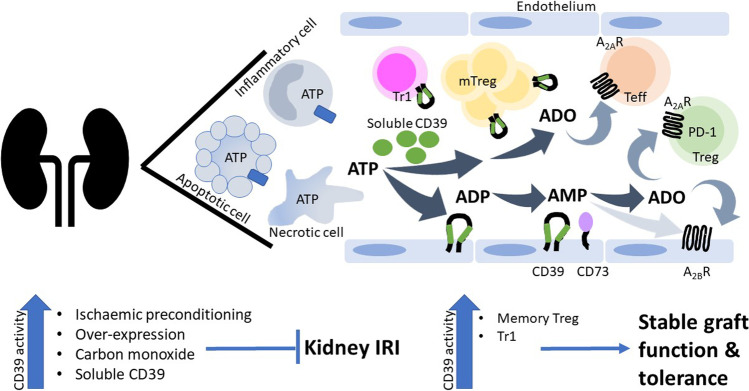
CD39-rich milieu improves outcomes in kidney transplantation. In IRI, ATP is released from inflammatory and apoptotic cells via connexin and pannexin hemi-channels (blue rectangle) or directly from necrotic cells into the extracellular space. ATP is converted through an enzymatic process by CD39 and CD73 on the endothelium to adenosine (ADO). Adenosine mediates its anti-inflammatory effects via A_2A_ receptor (A_2A_R) on T regulatory cells (Treg) and T effector cells (Teff) and A_2B_ receptor (A_2B_R) on the endothelium. CD39 activity may be increased by ischaemic preconditioning, over-expression, carbon monoxide or delivery of soluble CD39. Improved kidney transplant outcomes occur with greater circulating numbers of Tr1 and memory Treg (mTreg), both of which express CD39

Kidney transplantation is the preferred treatment for individuals with kidney failure offering improved quality and quantity of life. Despite significant advancements in short term graft survival, longer term survival rates have not improved greatly mediated in large by chronic antibody mediated rejection. Strategies to reduce the donor kidney antigenic load may translate to improved transplant survival. CD39 on the vascular endothelium and on circulating cells, in particular Treg, is upregulated in response to hypoxic stimuli and plays a critical role in regulating the immune response removing proinflammatory ATP and generating anti-inflammatory adenosine (Fig. 1). Augmenting CD39 in the peri-transplant period can mitigate IRI and high expression of CD39 on mTreg may promote kidney transplant tolerance.

## Data Availability

Upon request.
